# Intrauterine Intussusception Leading to Ileal Atresia in a Premature Baby

**DOI:** 10.21699/ajcr.v8i2.567

**Published:** 2017-03-18

**Authors:** Sirajuddin Soomro, Sikandar Ali Mughal, Fida Hussain Depar

**Affiliations:** 1 Department of Paediatric Surgery, Chandka Medical College Larkana, Pakistan; 2 Department of Anaesthesiology, Chandka Medical College Larkana, Pakistan

**Dear Sir**

Intussusception is relatively common in infants but occurs only 0.3% of cases during the first month of life.[1] Majority of neonatal intussusceptions occur in full term babies and and it is rare in the premature newborns. Poynter suggested intrauterine intussusception as a cause of intestinal atresia in 1922. Less than 100 cases of intrauterine intussusception in association with ileal atresia have been reported in the literature.[2,3] We are adding another case with similar anomaly.

A 5-day-old male baby, delivered through normal vaginal delivery around 36th week of gestation and weighing 1.7kg, presented with failure to pass meconium, bilious vomiting, and distention of abdomen soon after birth. Antenatal history was unremarkable. On examination patient was dehydrated and abdomen found distended. Per rectal examination showed normal size, shape and location of anus and empty rectum. On x-ray abdomen multiple air and fluid levels found. X-Ray gastrografin enema showed unused colon. After resuscitation, patient underwent exploratory laparotomy. The loops of jejunum and ileum found distended proximal to an ileo ileal intussusception (Fig.1). The intussusception was reduced which revealed type II ileal atresia (Fig. 1). The dilated part of ileum and area of ileum containing atresia was resected. The tip of reduced intussusception found mummified and resembling meconium pellet like structure. Primary ileo ileal end to end anastomosis performed. Postoperative course was uneventful and patient discharged home on 9th postoperative day. Follow up revealed a growing healthy baby.

**Figure:**
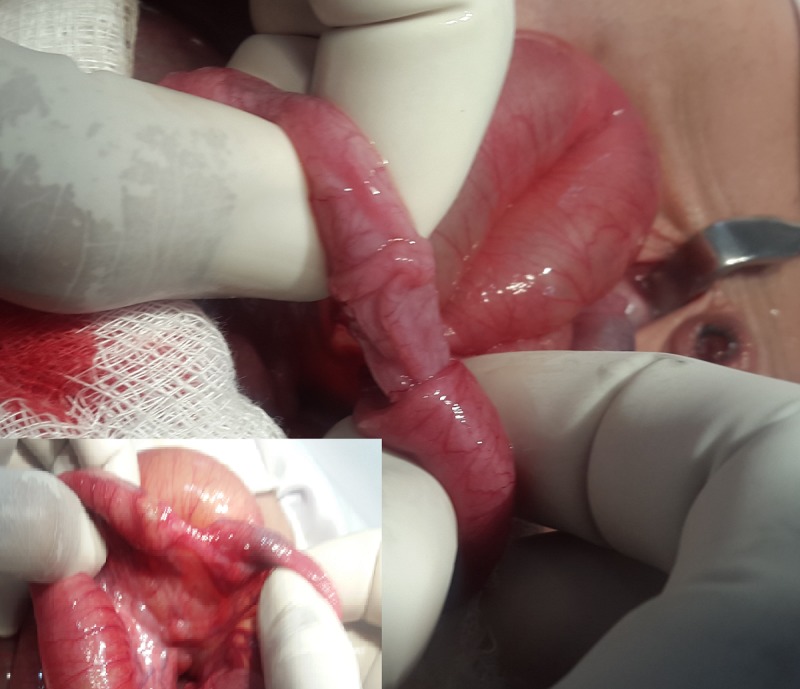
Figure 1: Ileo-ileal intussusception. Inset shows type II ileal atresia.

Intrauterine intussusception is estimated to cause 0.6% to 13.1% of intestinal atresias. The intussusception induced atresias tend to occur in ileum or jejunum and atresia with a mesenteric gap (type IIIa atresia) being common as compared to fibrous connecting cord type (type II atresia).[3] In our case the intussusception was in the ileum and type II atresia.

Antenatal intussusception can rarely be identified on antenatal ultrasound examination.[4] In our case the antenatal ultrasound was reported as normal. Intrauterine intussusception leading to ileal atresia is one of the rare causes of intestinal obstruction in the newborn period. The definitive diagnosis can only be established at the time of surgery.

## Footnotes

**Source of Support:** Nil

**Conflict of Interest:** None declared

